# Venous Thromboembolism Therapy with Apixaban in Daily Care Patients: Results from the Dresden NOAC Registry

**DOI:** 10.1055/s-0041-1728675

**Published:** 2021-05-04

**Authors:** Jan Beyer-Westendorf, Sandra Marten, Luise Tittl, Christiane Naue, Martin Bornhäuser

**Affiliations:** 1 Thrombosis Research Unit, Division Hematology, Department of Medicine I, University Hospital “Carl Gustav Carus” Dresden, Dresden, Germany; 2 Kings Thrombosis Service, Department of Hematology, Kings College, London, United Kingdom

**Keywords:** anticoagulation, bleeding, persistence, apixaban, venous thromboembolism

## Abstract

The effectiveness and safety of venous thromboembolism (VTE) treatment with apixaban, demonstrated in phase III trials, need to be confirmed in daily care.

Using data from the prospective, noninterventional cross-indication *Dresden NOAC Registry* we evaluated rates of VTE recurrence and bleeding complications during apixaban treatment of VTE patients. For this analysis, we only included patients with acute VTE who started apixaban within 14 days after diagnosis and who were enrolled within these 14 days. Patient characteristics, treatment persistence, and clinical outcomes were assessed.

Between August 1st, 2014 and October 31, 2018, 352 patients with apixaban treatment for acute VTE were enrolled. During treatment (median exposure 13.7 ± 9.8 months; median follow-up 21.7 ± 6.1 months) rates of recurrent VTE and International Society on Thrombosis and Haemostasis major bleeding were 1.3/100 pt.years (95% confidence interval or CI 0.4–3.0) and 1.5/100 pt.years (0.6–3.3), respectively. At 6 months. 68.6% of patients were still taking apixaban, 23.9% had a scheduled end of treatment, 6.3% were switched to other anticoagulants, and the remaining 2.3% had unplanned complete discontinuation of anticoagulation.

Of the 188 patients stopping apixaban, 12 (6.4%) experienced a recurrent VTE (six pulmonary embolisms ± deep vein thrombosis, six deep vein thrombosis; mean time between stopping anticoagulation and VTE recurrence 5.2 ± 4.1 months [range 14–417 days]).

Our findings suggest that, in daily care, apixaban demonstrated high effectiveness, safety, and persistence in the treatment of acute VTE with low rates of unplanned discontinuation.

## Introduction

After decades of VTE therapy with vitamin K antagonists (VKAs), nonvitamin K antagonizing oral anticoagulants (NOACs) have replaced VKA in this indication, following successful completion of large phase III trials some 10 years ago. 1 The main benefits of NOAC include a much more predictable dose–response relationship, limited interactions with co-medications and, therefore, a better convenience for patients who no longer require routine monitoring or frequent dose adjustments. 2 3

Apixaban is a direct factor Xa inhibitor that has been approved for the treatment of deep vein thrombosis (DVT) and pulmonary embolism (PE), following the finalization of two large phase III trials (AMPLIFY and AMPLIFY EXTENSION) 4 5 and, more recently, the cancer VTE study CARAVAGGIO. 6

In AMPLIFY, apixaban was tested against low molecular weight heparin (LMWH) followed by VKA for the treatment of acute DVT and/or PE. Of note, in this study only patients with unprovoked VTE or provoked VTE with persisting risk factors were included and patients were excluded if they received other anticoagulants for initial VTE therapy for more than 36 hours. In this study, apixaban demonstrated noninferiority in efficacy and superiority for safety in comparison to LMWH/VKA.

In the three-armed AMPLIFY EXTENSION trial, two doses of apixaban (continued therapeutic dosage of 5 mg BID vs. prophylactic dosage of 2.5 mg BID) were compared against placebo in VTE patients who had completed a 6 to 12 months course of anticoagulation for acute DVT and/or PE and in whom uncertainty existed whether anticoagulation should be continued or stopped at this point in time. In this trial, both apixaban dosages drastically reduced the risk of recurrent VTE (relative risk reduction 64 and 67% compared with placebo) without increasing the risk of major bleeding.

Based on these results, apixaban has been approved and rapidly adopted in many countries and current guidelines list apixaban as one of the preferred options for acute and long-term VTE treatment. 2 3

However, as with all phase III trials, the question of external validity arises, since these trials apply rigorous inclusion and exclusion criteria, regulate all medical interventions around the target disease, and apply close monitoring and strict outcome assessments, ensuring maximal patient safety and data quality. 7 At the same time, these prespecifications may limit generalizability of trial data to the daily care management of patients with acute or chronic VTE. Therefore, observational studies are requested by physicians and authorities to complement phase III trial findings. Furthermore, such observational studies can supplement phase III trial data by evaluating patient selection patterns, outcome management, and treatment persistence to anticoagulation, all of which is difficult to evaluate in a randomized setting that applies strict prespecified management recommendations.

With this in mind, we set up the prospective multicentric cross-indication *DRESDEN NOAC REGISTRY* in 2011, which is still ongoing. Here, we report the collected data on acute VTE management with apixaban.

## Methods

### Patients

The *DRESDEN NOAC REGISTRY* (NCT01588119) is a prospective registry in the administrative district of Dresden (Saxony), Germany, covering a network of more than 230 enrolling physicians from private practices and hospitals. Patients are eligible if they receive at least 3 months of NOAC anticoagulation for any indication and are willing to participate in and available for quarterly follow-up visits by phone, performed by the central registry office. No exclusion criteria apply. The design and methodology of the Dresden NOAC Registry has been published previously. 8

As a noninterventional study, patients are not actively examined for recurrence. In case of suspected outcome events, medical reports are requested from health care providers and presented to an adjudication committee. If medical reports are inconclusive, the worst-case approach is applied.

The protocol does not include a formal adjudication of the index DVT/PE, since diagnosis of acute VTE in Germany is predominantly in the hands of vascular or cardiac specialists and objective testing (mainly with ultrasound for suspected DVT and computed tomography pulmonary angiography or V/Q scan for suspected PE) is readily available. The established standard of care is consistently used across Germany, as has been demonstrated by the TULIPA registry. 9

For the presented analysis, only patients with acute PE and/or acute distal or proximal lower limb DVT who started apixaban within 14 days after diagnosis of VTE and who were enrolled within these 14 days were evaluated with regard to patient characteristics, treatment persistence, and clinical outcomes.

### Outcome Measures

To assess effectiveness of apixaban therapy in VTE, the annualized rate of the recurrent VTE was evaluated. Cases of sudden death were adjudicated as fatal PE and counted as a recurrent VTE event if other causes of death were not established, and PE could not be ruled out.

The main safety outcome was the annualized rate of major bleeding according to the International Society on Thrombosis and Haemostasis (ISTH) definition. 10 Further safety outcomes were rates of ISTH nonmajor clinically relevant (NMCR) bleeding, minor bleeding, and all-cause mortality.

Crude outcome numbers are reported for days 90, 180, 365, and >365 and annualized event rates for 180 and 365 days.

To put outcome event rates into perspective with available real-world data in the discussion section, a formal literature search was performed on February 15, 2020 using the search terms “apixaban” in combination with “deep vein thrombosis or DVT,” “pulmonary embolism or PE,” “venous thromboembolism or VTE,” and “real world.”

### Treatment Discontinuation

In accordance with previously published analyses from our registry, 11 treatment discontinuation was defined as a permanent discontinuation or an unscheduled interruption of apixaban for longer than 4 weeks without the initial plan to restart apixaban. This included patients who were permanently switched to another anticoagulant. In contrast, treatment persistence was defined as the continuation of apixaban therapy over the entire follow-up period, allowing for temporary interruptions. At every visit, any change in anticoagulant therapy is assessed. Reasons for switching to other anticoagulants or stopping anticoagulation as well as the future treatment plan were obtained from patients or attending physicians. Missing values were left blank and not replaced by imputation.

### Statistics

Two different analysis sets were defined and evaluated:

1. The overall rate of recurrent VTE was evaluated in the *intention-to-treat analysis,* including all VTE patients who were enrolled in the registry and received apixaban for acute VTE at baseline. In this analysis, all effectiveness outcome events were included that occurred throughout the follow-up period, despite the anticoagulation status.
2. Rates of recurrent VTE and bleeding complication (all, major, and NMCR) during the time of anticoagulation therapy (and up to 3 days after last intake) were evaluated in the on-treatment analysis. Temporary treatment interruptions (up to 3 days) were tolerated.

Data analysis was conducted according to a predefined statistical analysis plan. Baseline characteristics are presented as absolute and relative frequencies, mean and standard deviation, or median with interquartile range, where appropriate. Annualized event rates (per 100 patient years) were calculated using Kaplan–Meier time-to-first-event analysis and reported with the corresponding 95% confidence intervals (CIs). Due to the small sample size, comparative statistics of subgroups were omitted.

In addition, the following sensitivity analyses were performed as Kaplan–Meier time-to-first event analyses:

- Recurrent VTE during the acute phase until day 90 for patients who started apixaban within 72 hours; 3 to 7 days or 8 to 14 days after VTE diagnosis.
- Recurrent VTE during the acute phase until day 90 for patients who were treated for provoked VTE by major transient trigger versus minor transient or persistent trigger versus unprovoked VTE. Major transient triggers included complete immobilization for at least 3 days or major surgery within 4 weeks prior to VTE diagnosis or active cancer. Surgery was deemed “major” if general anesthesia for greater than 30 minutes was performed. 12 Minor triggers included long-distance travel, acute infectious diseases without immobilization, estrogen use, obesity with BMI >30, pregnancy or puerperium, family history of VTE.
- Net clinical benefit was observed (defined as recurrent VTE and/or major bleeding and/or all-cause mortality) for patients who had a scheduled end of treatment at 6 months and stopped apixaban between days 150 and 210 versus those who were selected to continue apixaban beyond 210 days (who, in case of later treatment cessation, were censored at the day of apixaban discontinuation).

All statistical analyses were performed using the IBM SPSS Statistics version 25, MedCalc version 14.8.1.

## Results

**Fig. 1:**
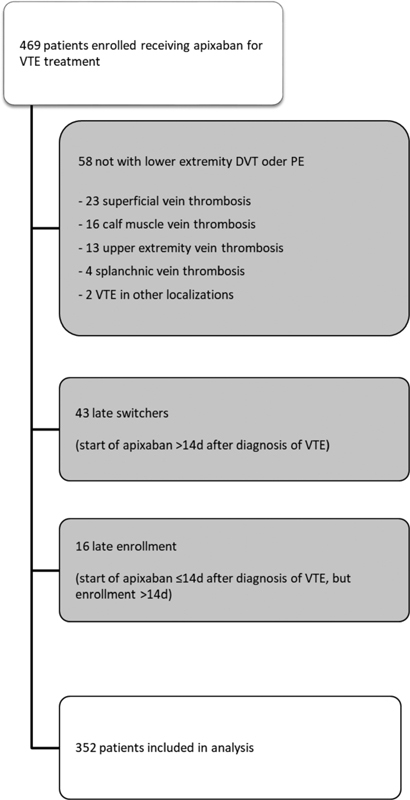
Flowchart of study cohort.

Between December 1st, 2011 and October 31, 2018, a total of 4,385 patients were enrolled into the *DRESDEN NOAC REGISTRY.* Of these, 469 were receiving apixaban for VTE treatment and 352 (75.1%) fulfilled the selection criteria for the present analysis. Reasons for exclusion are demonstrated in Fig. 1 .

The VTE index event was an isolated DVT without confirmed PE in 276 (78.4%) cases and 76 (21.6%) patients had an objectively confirmed PE with or without DVT. Overall, 51.4% were male and median age was 66 years (IQR 25 years). Details on patient characteristics and index VTE are presented in Table 1 . Mean time between VTE diagnosis and initiation of apixaban was 1.0 ± 2.7 days (median 0 days; IQR 0 days) and numerically longer for PE versus DVT (mean 2.9 ± 3.8 days vs. 0.5 ± 1.9 days). At baseline, apixaban was prescribed at a dose of 10 mg BID in 90.9%, 5 mg BID in 7.1%, and 2.5 mg BID in 2.0% of patients. Reasons for not using 10 mg apixaban BID initially were pretreatment with therapeutic parenteral anticoagulants for ≥7 days in 20 cases, comorbidities (e.g., bleeding history, renal impairment) in seven cases, and not reported in the remaining five cases.

**Table 1:** Patient characteristics at baseline

	All *n* = 352	DVT *n* = 276	PE ± DVT *n* = 76
Male, *n* (%)	181 (51.4)	136 (49.3)	45 (59.2)
Median age (IQR), years	66.0 (25.0)	66.5 (25.0)	65.5 (23.0)
Number of concomitant drugs, mean (SD)	3.0 ± 3.0	2.8 ± 2.9	4.1 ± 3.2
Mean time between VTE diagnosis and initiation of apixaban (SD), days	1.0 ± 2.7	0.5 ± 1.9	2.9 ± 3.8
Unprovoked VTE, *n* (%)	118 (33.5)	87 (31.5)	31 (40.8)
Event VTE provoked by minor persistent or transient triggers, *n* (%)	173 (49.1)	136 (49.3)	37 (48.7)
Event VTE provoked by major transient triggers, *n* (%)	61 (17.3)	53 (19.2)	8 (10.5)
Recurrent VTE, *n* (%)	82 (23.3)	65 (23.6)	17 (22.4)
Proximal vs. distal DVT, *n* (%)	197 (56.0) vs. 79 (22.4)	197 (71.4) vs. 79 (28.6)	n.a.
Malignant disease, *n* (%)	42 (11.9)	33 (12.0)	9 (11.8)
• Active cancer, *n* (%)	5 (1.4)	1 (0.4)	4 (5.3)
Glomerular filtration rate (GFR) <50 mL/min, *n* (%)	31 (8.8)	23 (8.3)	8 (10.5)
• GFR 30–50 mL/min, *n* (%)	29 (8.2)	21 (7.6)	8 (10.5)
• GFR <30 mL/min, *n* (%)	2 (0.6)	2 (0.7)	0

Abbreviations: DVT, deep vein thrombosis; GFR, glomerular filtration rate; IQR, interquartile range; PE, pulmonary embolism; SD, standard deviation; VTE, venous thromboembolism.

During a mean follow-up (FU) of 21.7 ± 6.1 months (median 24 months; IQR 5.2; range 31–1,114 days), a total of 17 patients (4.8%) experienced a recurrent VTE, which translated into a recurrence rate of 2.8/100 patient years (pt. years; 95% CI 1.6–4.4) for the intention-to-treat population.

The mean time of apixaban exposure was 13.7 ± 9.8 months (median 12.4 months; IQR 20.4) and, during active treatment with apixaban, five patients experienced a recurrent VTE. This translated into a recurrence rate of 1.3/100 pt. years (95% CI 0.4–3.0) for the on-treatment population. VTE recurrence rates were highest during the first 90 days of therapy ( Table 2 ; Supplementary Fig. S1 ).

**Table 2:** Outcome event rates according to treatment phase and treatment continuation

*n* = 352	Events 0–90 d		Events 91–180 d		Events 181–365 d		Events >365 d	
	ITT	On treatment	ITT	On treatment	ITT	On treatment	ITT	On treatment
Recurrent VTE; *n* (%)	3 (0.9)	2 (0.6)	1 (0.3)	0	5 (1.4)	0	8 (2.3)	3 (0.9)
Fatal VTE; *n* (%)	0	0	0	0	0	0	0	0
Major bleeding; *n* (%)	3 (0.9)	0	2 (0.6)	0	4 (1.1)	3 (0.9)	6 (1.7)	3 (0.9)
Fatal bleeding; *n* (%)	0	0	1 (0.3)	0	0	0	0	0
Mortality; *n* (%)	1 (0.3)	0	3 (0.9)	0	2 (0.6)	0	6 (1.7)	1 (0.3)

Abbreviations: ITT, intention-to-treat population, which includes all outcome events during follow-up, irrespective of anticoagulation status; VTE, venous thromboembolism.

A total of 124 patients (35.2%; 43/100 pt. years; 95% CI 35.7–51.2) reported any bleeding events during apixaban exposure. ISTH major bleeding occurred in six cases (1.5/100 pt. years; 95% CI 0.6–3.3), including three intracranial bleedings, two cases of genitourinary bleeding, and one case of postoperative bleeding with drop in hemoglobin >2 g/dL. Furthermore, NMCR bleeding events occurred in 55 cases (15.6%; 15.3/100 pt. years; 95% CI 11.6–20.0), predominantly manifesting as skin/mucosal bleeding (61.8%), genitourinary bleeding (20.0%), gastrointestinal bleeding (12.7%), or other bleeding types (5.5%).

During apixaban treatment, rates for recurrent VTE were numerically lower in patients with DVT as an index event (1.1/100 pt. years; 95% CI 0.2–3.1) compared with PE as an index event (1.8/100 pt. years; 95% CI 0.2–6.5) and similar trends were observed for rates of major bleeding (1.1/100 pt. years for index DVT; 95% CI 0.2–3.1 vs. 2.7/100 pt. years for index PE; 95% CI 0.6–7.8). Effectiveness and safety profiles were consistent across relevant subgroups ( Supplementary Table S1 and Supplementary Fig. S2 ). Furthermore, in another sensitivity analysis we did not detect a signal that early (within 2 days after VTE diagnoses) or delayed (8–14 days after diagnosis) start of apixaban therapy correlated to an increased risk of VTE recurrence ( Supplementary Fig. S3 ).

A total of 12/352 patients died (1.9/100 pt. years; 95% CI 1.0–3.3) of which one death occurred during or within 3 days after last intake of apixaban (0.25/100 pt. years; 95% CI 0–1.4). Causes of death were cancer ( *n* = 5), fatal cardiovascular event ( *n* = 2 including one case of sudden cardiac death and one case of congestive heart failure), sepsis/infection ( *n* = 2), age-related death ( *n* = 2), and one case of fatal bleeding unrelated to apixaban exposure.

At 6 months (FU completed in 352 pts.), 341 patients (96.9%) were still alive. Of these, 69.8% of patients were still taking apixaban, 24.6% had a scheduled end of treatment, 6.5% were switched to other anticoagulants, and the remaining 2.3% had unplanned complete discontinuation of anticoagulation.

At 12 months (FU completed in 328 pts.), 316 patients (96.3%) were still alive. Of these, 57.9% of patients were still taking apixaban. The remaining patients having a scheduled end of treatment (33.5%), were switched to other anticoagulants (8.5%) or had an unplanned complete discontinuation of anticoagulation (3.8%).

Of the 188 patients stopping apixaban, 12 (6.4%) experienced a recurrent VTE (six PE ± DVT, six DVT) and the mean time between stopping anticoagulation and VTE recurrence was 5.2 ± 4.1 months. Despite discontinuation of oral anticoagulation in a total of 188 patients, major bleeding still occurred in nine patients (4.8%; Supplementary Table S2 ), which translated into an anticoagulation-unrelated major bleeding rate of 3.9/100 pt. years (95% CI 1.8–7.5), indicating that the major driver for bleeding is patient-related and not anticoagulation-related, which likely contributed to the decision to stop anticoagulation.

## Discussion

In a large cohort of patients treated with apixaban for acute VTE in daily care, we could demonstrate a general confirmation of the effectiveness and safety of apixaban demonstrated in phase III studies. In the AMPLIFY phase III trial which did not report annualized rates, absolute rates of recurrent VTE and ISTH major bleeding were 2.3 and 0.6%, respectively for patients treated for 6 months. 4 5 In our intention-to-treat analysis, recurrent VTE occurred in 4.8% of patients (2.8/100 pt. years) and ISTH major bleeding in 4.3% of patients (2.4/100 pt.years). The higher event rates seen in our study may partly be explained by the fact that no exclusion criteria were applied in our cohort of consecutive daily care patients, whereas phase III trials prohibit enrolment of patients at unacceptably high risk for complications by defining explicit inclusion and exclusion criteria. Furthermore, mean age of our cohort (66 years) was considerably higher than that in the AMPLIFY (mean 57 years). 4 In addition, the majority of patients in AMPLIFY were patients with unprovoked VTE (excluding patients with VTE shortly after major trauma or surgery, which may carry a higher risk for VTE recurrence or bleeding especially in the early phase of anticoagulation), whereas we included a total of 61 (17.3%) patients with VTE provoked by a major transient risk factor such as recent surgery or trauma. Numerically, these patients presented slightly higher rates of VTE recurrence (1.6 vs. 0.8% in patients with unprovoked VTE; Supplementary Table S1 ) but the absolute difference was small and based on low numbers. Reassuringly, rates for major bleeding were low in both cohorts. Another difference between AMPLIFY and our cohorts' study was the length of observation (6 vs. 21 months). Therefore, neither our crude bleeding incidences nor our annualized bleeding event rates, derived from a cohort with considerably longer treatment and follow-up duration, can be directly compared with the phase III data. However, at 6 months, our crude event rates of 1.1% (recurrent VTE) and 1.4% (major bleeding; Table 2 ) were not far from those reported in AMPLIFY.

In addition to thromboembolic and bleeding outcomes we reported on treatment persistence and reasons for discontinuation. We found a reassuringly low rate of unscheduled premature discontinuation (2.3% at 6 and 3.7% at 12 months) and a clear trend toward prolonged anticoagulant treatment with apixaban, since nearly 70% of patients were treated for longer than 6 months. The low rate of unscheduled premature discontinuation is reassuring and an indicator of high patient and physician satisfaction. The trend to prolonged treatment is in line with the pronounced risk profile for VTE recurrence in our cohort and, thus, in line with current guideline recommendations.

Our data are not the first real-life data for VTE treatment with apixaban. Several large retrospective health record or claims data analyses from Denmark and the United States have been published. 13 14 15 16 17 Although all these studies have evaluated much larger cohorts of VTE patients, by definition they are retrospective in nature, mostly rely on ICD codes, and apply algorithms to identify potential outcome events which are not centrally adjudicated. As such, there are many known and unknown confounders in these studies, which also lack granularity in details of patient selection, drug management, and reasons for treatment changes. For this, prospective registry data are needed and, to our knowledge, only one such prospective registry has been published do date. 18 In this prospective single center cohort of 300 apixaban patients, only those who completed 3 months of treatment or had an outcome event were evaluated, resulting in a total of 302 patients. Rates of recurrent event at 3 months was 2.3% and major bleeding occurred in 11 patients (3.6%), which is somewhat higher than our data, although no direct comparisons can be made, since treatment durations were different and no details on drug dosage or exposure time were reported. It has also to be noted that in this Mayo Clinic project, a total of 1,696 consecutive patients were enrolled, of which only 17.8% received apixaban, which indicates a high degree of dedicated patient selection. Strikingly, this group reported surprisingly low rates of NMCR bleeding (2.3%) which is not in line with our data (15.6%) or those collected in AMPLIFY, raising the question for a potential of under-reporting of nonmajor bleeding events in their study.

In contrast, our study reports on a cohort of apixaban patients recruited at multiple centers and we followed all patients (irrespective of premature treatment discontinuation) for up to 2.5 years. We reported outcome events that occurred during active treatment and after discontinuation and provided outcome data based on central event adjudication. Our data also did not indicate that early start of apixaban or prolonged alternative (usually LMWH) therapy for up to 2 weeks before apixaban initiation leads to different rates in VTE recurrence, which is also reassuring, since AMPLIFY did not allow for a pretreatment with LMWH for more than 36 hours. Furthermore, we assessed treatment persistence and reasons for treatment changes, all of which have not been reported so far. To put our findings into perspective with the abovementioned reports we have summarized the main design items and results of published apixaban VTE cohorts in Table 3 . However, this is meant to be descriptive only and readers are cautioned not to compare these very different studies directly. Despite the differences in patient characteristics and outcome assessments, our data are very much in line with most of the outcome data in previous real-world studies, which indicates that apixaban real-world evidence confirms the effectiveness and safety of apixaban in VTE treatment across a spectrum of very different methodologies, somewhat different populations, and outcome event definitions.

**Table 3:** Available real-word data on the effectiveness and safety of VTE treatment with apixaban

Study	Study design	Central event adjudi-cation	Data on apixaban persistence	Data on apixaban dosing	Outcome data after stopping apixaban	Apixaban initiation in acute phase	Sample size apixaban cohort	Mean age	Pro-portion of PE	Unpro-voked VTE	Cancer	Duration of follow-up	VTE recurrence during apixaban treatment	Major bleeding during apixaban treatment
Present study	Prospective multicenter registry	Yes	Yes	Yes	Yes	Yes	352	66 y	21.6%	33.5%	11.9%	21.7 mo	1.4% (1.3/100 pt. years)	ISTH: 1.7% (1.5/100 pt. years)
Bott-Kitslaar et al 18	Prospective single center registry	Yes	Yes	Ø	Ø	Yes	302	62.4 y	38.7%	16.6%	47.0%	3 mo	2.3%	ISTH: 3.6%
Haastrup et al 16	Retrospective health record analysis	Ø	Ø	Yes	Ø	All first prescriptions (incl. nonacute VTE)	2,346	Unk	Unk	Unk	Unk	Unk	Unk	Unk
Lutsey et al 13	Retrospective claim database analysis, U.S. MarketScan	Ø	Ø	Ø	Ø	All first prescriptions (incl. nonacute VTE)	4,128	56.8 y	Unk	Unk	Unk	Unk	Unk	Unk
Sindet-Pedersen et al 23	Danish National Patient Register	Ø	Ø	Ø	Ø	Yes	1,504	Median: 70 y	53.5%	Unk	14%	Median: 180 d	Standardized 180 d risk 2.16%	Standardized 180 d risk 1.73%
Dawwas et al 14	Retrospective claim database analysis Truven, Medicare	Ø	Ø	Ø	Ø	All first prescriptions (incl. nonacute VTE)	3,091	61.6 y	100%	50.9%	17.7%	99 d	3/100 pt. years	3/100 pt. years
Dawwas et al 15	Retrospective claim database analysis (MediCare and others)	Ø	Ø	Ø	Ø	All first prescriptions (incl. nonacute VTE)	8,094	58.9 y	Unk	47.5%	16.2%	Unk	1.6%	1.1%
Weycker et al 17	Retrospective U.S. claims database analysis	Ø	Ø	Ø	Ø	All first prescriptions (incl. nonacute VTE)	17,878	60.0 y	41.0%	77.2%	1.2%	143 d (estimated exposure 116 d)	2.3%	1.7% based on primary ICD codes

Abbreviations: ISTH, International Society on Thrombosis and Haemostasis; VTE, venous thromboembolism.

## Limitations

There are several limitations to our study, which have been discussed in detail in previous publications. 8 19 20 For the presented analysis, some specific limitations apply.

### Sample Size and Potential for Selection Bias

Our cohort consisted of 352 patients, and our sample size as well as the small number of outcome events may have been too small to detect clinically relevant differences, which is especially true for our descriptive subgroup analyses. The design of our registry introduces the possibility of a selection bias because local physicians within the network are not instructed on which of their patients should receive which type or dosage of oral anticoagulant therapy.

### Lack of Randomized Comparator

The lack of a direct randomized comparator group needs to be regarded as a limitation. However, many large observational VTE treatment studies exist from the VKA era 21 and data on VKA complications and VKA treatment discontinuation are well established. 22 In addition, health record and large claim database analyses have attempted to compare VTE treatment of apixaban with VKA 17 23 using matching procedures. Although this is certainly an important and valid statistical approach it should be noted that matching will not completely rule out residual confounding, also, because a relevant proportion of patients are often not eligible for matching. Therefore, also the inclusion of a comparator group will cause problems for data analysis and interpretation, as has been demonstrated in the XALIA registry. 24 25

Despite all potential limitations, in comparison to existing real-world data on apixaban VTE treatment, the long follow-up duration, the prospective data collection at patient level in daily care patients, and the central adjudication of effectiveness, and safety outcomes are significant advantages of our study.

## Conclusion

In daily care, apixaban treatment for acute VTE is effective and acceptably safe. We found initial apixaban dosing to be in accordance with label in over 90% of patients and, at 6 and 12 months, persistence was high with low rates of unplanned complete discontinuation. Fatal VTE and fatal bleeding are rare events during apixaban therapy and all-cause mortality is mostly related to underlying diseases, age, or acute conditions. Treatment discontinuation resulted in a relevant increase in VTE recurrence, of which 50% manifested as PE. Furthermore, bleeding complications were still frequently seen in patients stopping apixaban therapy indicating a selection pattern of stopping anticoagulation mainly in patients at high risk for (anticoagulation unrelated) bleeding despite high risk for VTE recurrence. Consequently, the concept of low dose apixaban therapy in secondary VTE prevention, established in AMPLIFY EXTENSION 5 needs further evaluation in real-world studies.

## Notes

**What Does This Paper Add? What Is Known About the Subject**

- Treatment of venous thromboembolism (VTE) with apixaban was effective and safe in the *AMPLIFY/AMPLIFY EXTENSION* trials. However, due to the strict selection criteria in phase III trials, post marketing data are needed to confirm the generalizability of trial data.
- So far, real-world data on apixaban treatment for acute VTE are limited, often derived from retrospective analyses and frequently lacking details on dosing, treatment persistence, or long-term outcomes.
- Especially data for apixaban use in the treatment of patients with VTE provoked by major transient risk factors are needed, since such patients were excluded from participation in AMPLIFY.

**What This Paper Adds**

- Data from ongoing, prospective, noninterventional *Dresden NOAC Registry* aimed to confirm the phase III trial results in daily care and to provide outcome data for up to 2 years.
- In daily care, acute venous thromboembolism (VTE) therapy with apixaban showed acceptably low VTE recurrence and major bleeding rates over a follow-up duration of 2 years. Adherence to dosing recommendations and treatment persistence were satisfactory.
- Apixaban demonstrated little differences in outcome rates for patients with provoked versus unprovoked VTE, confirming a broad generalizability of AMPLIFY trial data.
