# Supplementary material for: Venous Thromboembolism Therapy with Apixaban in Daily Care Patients: Results from the Dresden NOAC Registry
Source: TH Open. 2021 May 4;5(2):e143–51. doi: 10.1055/s-0041-1728675 (PMC8096533; doi:10.1055/s-0041-1728675)
Supplement: Supplementary file 1 — Supplementary Material [file 10-1055-s-0041-1728675-s200078.pdf]

Supplementary Table S1 Apixaban treatment duration and crude incidences of recurrent VTE or major bleeding, according to patient characteristics at baseline

	Mean treatment duration (d)	Crude incidence of recurrent VTE during treatment *n* (%)	Crude incidence of ISTH major bleeding during treatment *n* (%)
Total	409.9	5/352 (1.4)	6/352 (1.7)
Index VTE as PE (*n* = 76)	537.6	2/76 (2.6)	3/76 (3.9)
Index VTE as proximal DVT (*n* = 197)	450	2/197 (1)	2/197 (1)
Index VTE as distal DVT (*n* = 79)	187.2	1/79 (1.3)	1/79 (1.3)
Index VTE as unprovoked VTE (*n* = 118)	514.2	1/118 (0.8)	1/118 (0.8)
Index VTE provoked by minor persistent or transient triggers (*n* = 173)	410.2	3/173 (1.7)	5/173 (2.9)
Index VTE provoked by major transient triggers (*n* = 61)	207.4	1/61 (1.6)	0
Male (*n* = 181)	434	2/181 (1.1)	2/181 (1.1)
Female (*n* = 171)	384.4	3/171 (1.8)	4/171 (2.3)
Age ≥65 y (*n* = 189)	457.6	3/189 (1.6)	4/189 (2.1)
Age <65 y (*n* = 163)	354.6	2/163 (1.2)	2/163 (1.2)

Abbreviations: DVT, deep vein thrombosis; PE, pulmonary embolism; VTE, venous thromboembolism.

Supplementary Table S2 Types of recurrent VTE and major bleeding in patients during apixaban intake and after discontinuation

	On treatment (last intake ≤3 d)	Off treatment (permanently discontinuation)
Recurrent VTE		
Event/100 pt.years	1.3 (0.4–3.0)	5.4 (2.8–9.4)
n %	5/352 (1.4)	12/188 (6.4)
• Recurrence as PE	2	6
• Recurrence as DVT	3	6
ISTH major bleeding		
Event/100 pt.years	1.5 (0.6–3.3)	3.9 (1.8–7.5)
n %	6/352 (1.7)	9/188 (4.8)
• Gastrointestinal	0	3
• Genitourinary	2	1
• Intracranial	3[a1]	2
• Intraocular	0	1
• Other	1	2

Abbreviations: DVT, deep vein thrombosis; ISTH, International Society on Thrombosis and Haemostasis; PE, pulmonary embolism; VTE, venous thromboembolism.

 © 2021. The Author(s).

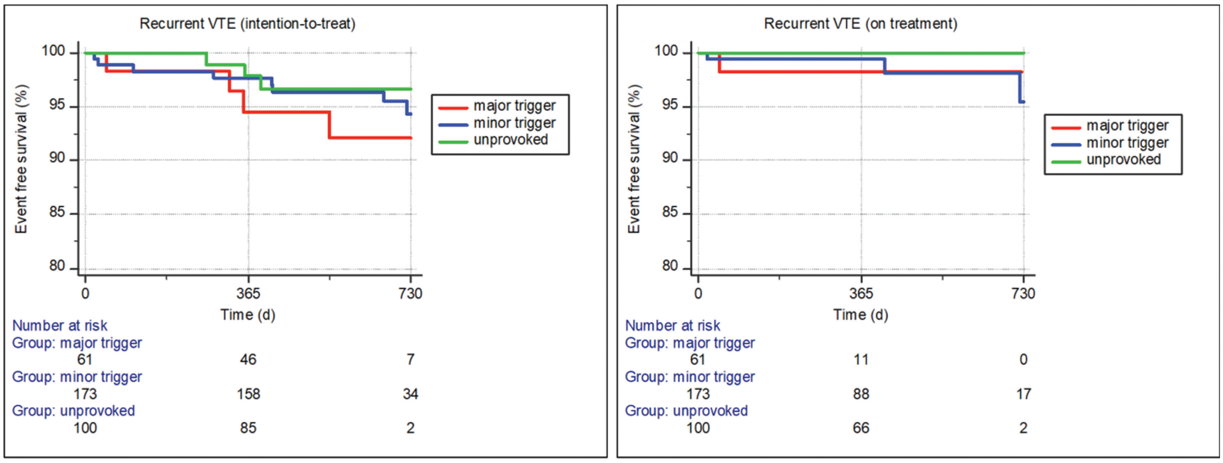

Supplementary Fig. S1 Recurrent VTE in the intention-to-treat analysis (*left panel*) and in the on-treatment analysis (*right panel*) according to the type of index event. VTE, venous thromboembolism.

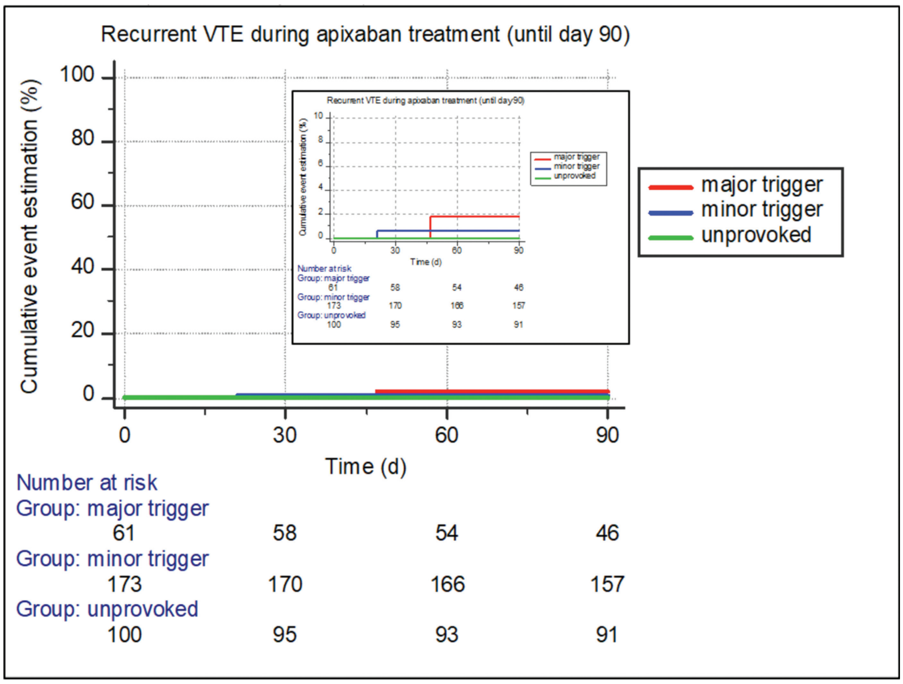

Supplementary Fig. S2 Recurrent VTE during the acute treatment phase (until day 90) in the on-treatment analysis according to the type of index event. VTE, venous thromboembolism.

 © 2021. The Author(s).

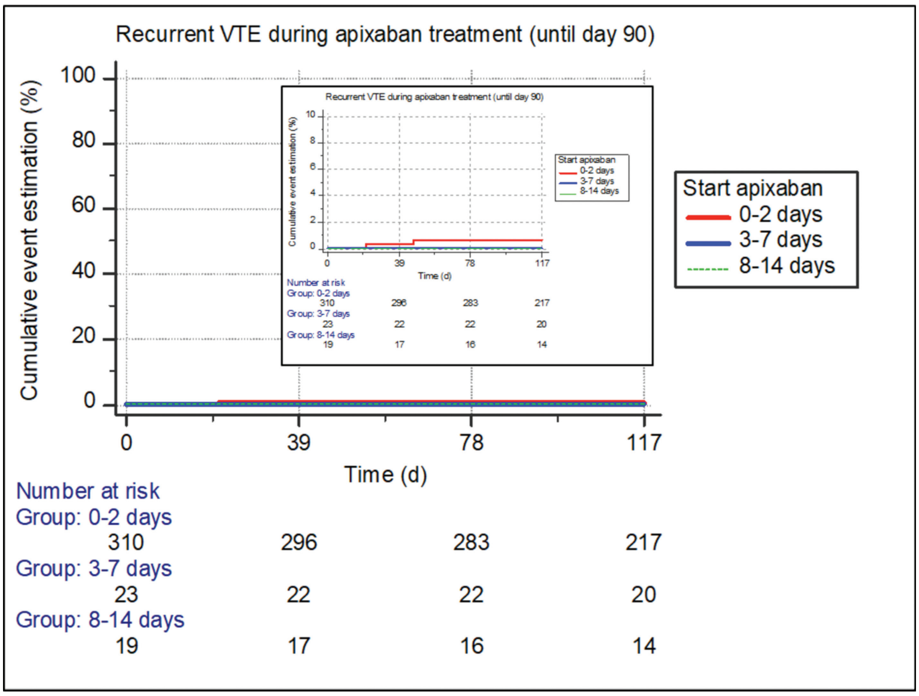

Supplementary Fig. S3 Recurrent VTE during the acute treatment phase (until day 90) in the on-treatment analysis according to time between VTE diagnosis and apixaban initiation (pretreatment with heparin was allowed for up to 14 d). VTE, venous thromboembolism.

 © 2021. The Author(s).
